# Study on the Effect of Electrochemical Dechlorination Reduction of Hexachlorobenzene Using Different Cathodes

**DOI:** 10.1155/2014/371510

**Published:** 2014-06-04

**Authors:** Yingru Wang, Xiaohua Lu

**Affiliations:** ^1^Environmental Science Research Institute, School of Environmental Science and Engineering, Huazhong University of Science and Technology, Wuhan 430074, China; ^2^School of Chemistry and Environmental Engineering, Wuhan Institute of Technology, Wuhan 430074, China

## Abstract

Hexachlorobenzene (HCB) is a persistent organic pollutant and poses great threat on ecosystem and human health. In order to investigate the degradation law of HCB, a RuO_2_/Ti material was used as the anode, meanwhile, zinc, stainless steel, graphite, and RuO_2_/Ti were used as the cathode, respectively. The gas chromatography (GC) was used to analyze the electrochemical products of HCB on different cathodes. The results showed that the cathode materials significantly affected the dechlorination efficiency of HCB, and the degradation of HCB was reductive dechlorination which occurred only on the cathode. During the reductive process, chlorine atoms were replaced one by one on various intermediates such as pentachlorobenzene, tetrachlorobenzene, and trichlorobenzene occurred; the trichlorobenzene was obtained when zinc was used as cathode. The rapid dechlorination of HCB suggested that the electrochemical method using zinc or stainless steel as cathode could be used for remediation of polychlorinated aromatic compounds in the environment. The dechlorination approach of HCB by stainless steel cathode could be proposed.

## 1. Introduction

Hexachlorobenzene (HCB) is a chlorinated compound. It is a common additive in agricultural antiseptics and also an intermediate in synthesis of organic chemicals. It has been used widely as a fungicide and an industrial synthetic material before its analogues were banned several decades ago. The chronic toxicity of HCB results in endocrine disruption, reproduction decrease, immune dysfunction, and neurobehavioral disorder [1, 2], which seriously damaged human health and attracted global concerns [3, 4], whereas HCB has been listed as one of the 12 priority persistent organic pollutants (POPs) for a global phase-out according to the Stockholm Convention on Persistent Organic Pollutants [5]. Due to its bioaccumulation, long-rang transport, and persistence, HCB has been observed all over the world in wastewater and sludge, water and sediment of rivers and lakes, biota, soil, and so forth. Current treatments of HCB in the soil and sediment of contaminated sites mainly use surfactant (because surfactant can increase the solubility of HCB in water) solution for chemical washing, rendering HCB enter into the aqueous phase with the surfactants from the treated soil or sediment. It is therefore necessary to carry out subsequent treatment on the HCB-polluted effluent. So far, seldom reports were published on the treatment of the HCB-polluted effluent.

At present, the treatments for HCB are performed by incineration, chemical degradation, biodegradation, photocatalytic oxidation, and the electrochemical method [6–9]. The electrochemical method is now emerging as a mild and environment-friendly process for destruction of POPs, in which the hazardous material can be transformed into harmless products in a closed system, with no toxic emissions [10]. The electrochemical destruction of chlorinated organic compounds has been extensively studied in aqueous solutions with various electrode materials, mainly metals, graphite, and composites [11–15]. In some literature on electrochemical treatment of wastewater, researchers were concerned about the oxidation of organic pollutants on the anode, while the reduction behavior of cathode is often neglected. Several studies [13, 16–19] found that, under appropriate electrolysis conditions, organic substance can be reduced to small organic molecules on the cathode, suggesting that the cathodic reduction and degradation of organic matter should not be neglected.

The cathode materials include metals, metal oxides, metals and composites of their oxides, and graphite. Kumiko Miyoshi et al. [10] tried to use the electrode with titanium-based coatings as the cathode to perform the dechlorination treatment on 1,2,3-TCB. The results revealed that the dechlorination rate of electrode with RuO_2_/Pt/PdO coatings was 91%, while that of RuO_2_ was 59%. Thus, electrode materials with prominent catalytic properties toward the reductive dehalogenation of C–Cl bonds are required. Additionally, Pd and Pt as well as some high hydrogen overvoltage metals such as Ag, Zn, Cu, Fe, Pb, and Ni were used as electrocatalytic cathodes for the reductive dehalogenation of chlorinated organic compounds.

In this work, the simulated effluent of HCB contaminated soil was processed by an electrochemical approach. The optimal operation condition of electrochemical approach for degradation of HCB was studied. The degradation mechanism of HCB was investigated by using RuO_2_/Ti as anode and zinc, stainless steel, graphite, and RuO_2_/Ti as cathode, respectively, in a self-prepared electrolysis cell. The effect of different cathodes on electrochemical reduction of HCB and the degradation mechanism were also discussed.

## 2. Experimental Sections

### 2.1. Experimental Materials

HCB (99.0%) was purchased from Shanghai General Reagent Factory, China. Standard products of other series of chlorobenzenes were obtained from Sigma-Aldrich or Fluka. TX-100 (analytical purity, 99.0%) was obtained from Amersco 94. The pH of solution was measured using a pH Meter (Shanghai, Leici). Deionized water was used for preparation of solutions. All the other reagents were analytical grade and used without further purification.

### 2.2. Batch Electrolysis

Batch electrolysis was conducted in a self-made electrolytic cell [20] of 200 mL with RuO_2_/Ti as anode (42 cm^2^) and different materials as cathode (42 cm^2^). The distance between anode and cathode was 2 cm. A laboratorial direct current power supply with current-voltage monitor was employed to provide electric power. The simulated wastewater was synthetic with an initial HCB concentration of 300 *μ*g/L, concentration of 1.0 g/L for the supporting electrolyte (Na_2_SO_4_), and an initial pH of 3.0.

### 2.3. Analytical Methods

At regular time intervals, 5 mL of sample was drawn from the electrolysis cell to analyze the concentration of HCB and dechlorination products. 2 mL hexane was added to the vial to extract all chlorobenzenes. HCB and dechlorination products in the supernatant were analyzed by a Hewlett-Packard 6890 gas chromatography (GC) equipped with an electron capture detector (ECD) and a ZB-5 capillary column. The carrier gas was chromatographic grade nitrogen (N_2_ 99.999%). The oven temperature was heated from 100 to 150°C at the rate of 25°C min^−1^, to 180°C at 20°C min^−1^, then to 190°C at 40°C min^−1^, and finally to 240°C at 10°C min^−1^. The flow rate of carrier gas was 2.0 mL min^−1^. The injector and detector temperatures were 250 and 300°C, respectively. The split ratio was 2 and injection volume was 1 *μ*L.

## 3. Results and Discussions

### 3.1. Optimization of Operation Parameters

In order to achieve the maximal removal of HCB in the effluent by the electrochemical method, the operation conditions were first optimized with RuO_2_/Ti as anode and stainless steel as cathode. The effects of the applied voltage, initial pH, initial HCB concentration, electrolysis time, and the concentration of electrolyte on the removal of HCB were investigated. The results showed that the best removal rate of HCB was up to 60.3% after 3 h with initial HCB concentration of 300 *μ*g/L, pH was at 3.0, with electrolyte concentration of 1.0%, voltage of 6 V, and TX-100 as a solubilizing agent.

### 3.2. HCB Dechlorination Efficiencies by Different Cathodes

Although the applied voltage, initial pH, HCB concentration, electrolysis time, and the concentration of electrolyte all have effects on the removal rate of HCB, the materials employed as anode and cathode play an important role. Before a further study on the effects of electrode materials, a diaphragm was added in our self-made electrolysis cell to investigate whether the electrochemical process of HCB was mainly an anodic oxidation or a cathodic reduction process. In this process, the cathode compartment and the anode compartment of the electrolysis cell were separated by a cation-exchange membrane to prevent the generation of Cl_2_ caused by the oxidation of Cl^−^ under the HCB reduction on the anode surface. The HCB concentration in both compartments was the same. It was reported [21] that if chloride ion was present in the solution, chlorine was produced on the anode and immediately reacted with water to form hypochlorite, which was a strong oxidizing agent and would react with ammonia during electrolysis. In our experiment, the results showed that the HCB concentration in cathode compartment was reduced gradually, while the HCB concentration in the anode one was nearly invariable after being electrolysed for 3 hours. Hence, HCB cannot be oxidized by hypochlorite. The electrochemical process of HCB was mainly a cathodic reduction process.

**Figure 1:**
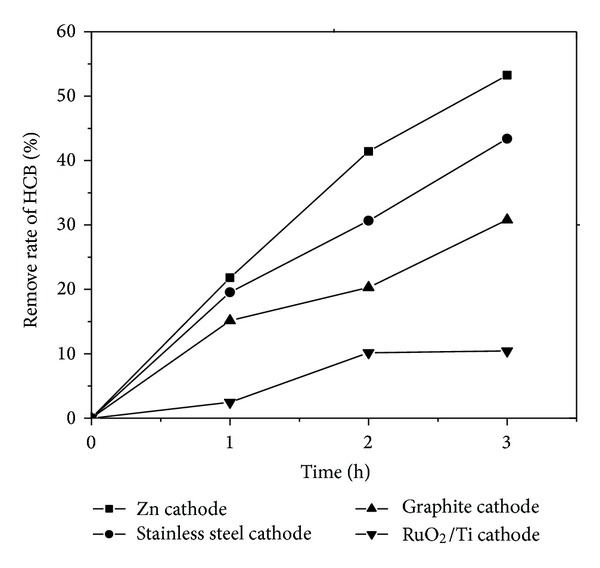
The removal rate of HCB with different cathode materials.

The effect of different cathodes on the removal efficiencies of HCB was examined by using RuO_2_/Ti as anode and Zn, stainless steel, graphite, and RuO_2_/Ti as cathode, respectively.  Figure 1 showed the removal efficiency of HCB with electrolysis time by using the four different cathode materials. As shown in Figure 1, the removal efficiency of HCB all increased with electrolysis time in the cathodic compartment, respectively. This trend could be attributed to the fact that hydrogen atoms were a powerful reducing agent for dechlorination, which could enhance the removal efficiency of HCB [22]. Moreover, the cathode materials significantly affected the removal efficiency of HCB. HCB exhibited the best effect with a removal efficiency of 53.2% after 3 h when zinc was used as the cathode, followed by stainless steel, of which removal efficiency of HCB was 43.4%. However, RuO_2_/Ti was not found to be capable of accelerating the cathodic reduction during the electrochemical treatment process of HCB. Instead, HCB showed the least effect when RuO_2_/Ti was used as the cathode with an HCB removal efficiency of only 10.4% after 3 h. Therefore, different cathode materials had different impact on the degradation of HCB. The descending order of removal efficiency was shown as follows: zinc > stainless steel > Graphite > RuO_2_/Ti.

In all, it could be concluded that zinc cathode was more suitable for HCB reduction than stainless steel, Graphite, and RuO_2_/Ti cathodes because of its high HCB reduction rate.

### 3.3. Analysis on the Intermediates of HCB Dechlorination with Different Cathode Materials

#### 3.3.1. Zinc and Stainless Steel Cathodes

**Figure 2:**
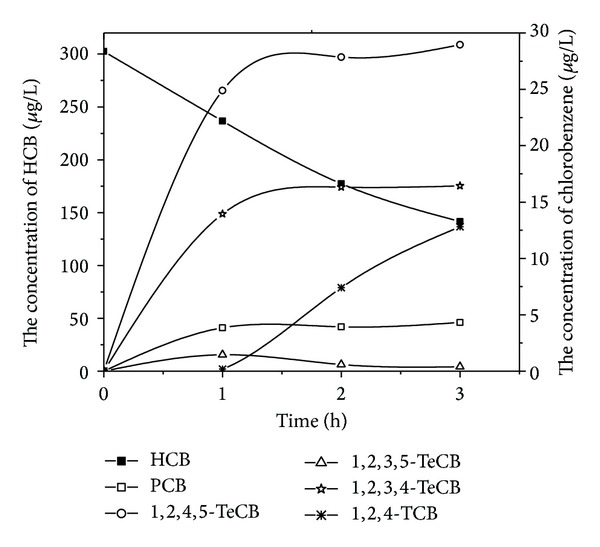
Variation of HCB and its dechlorination intermediates concentration using zinc cathode.

**Figure 3:**
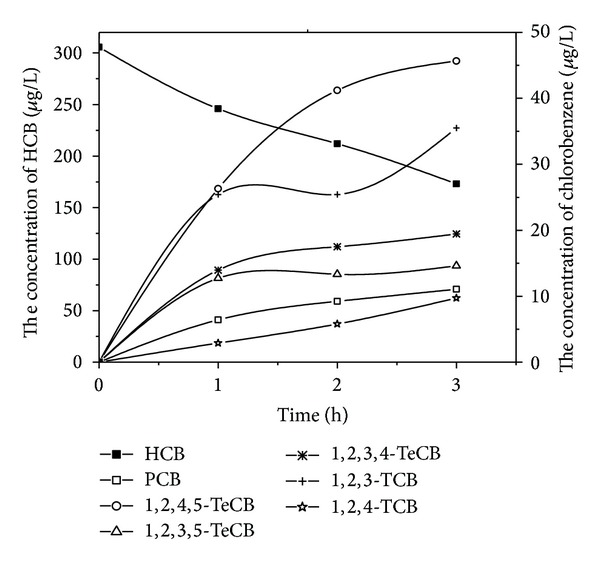
Variation of HCB and its dechlorination intermediates concentration using stainless steel cathode.

Figures 2 and 3 showed all detectable byproducts with time varying during the HCB electrochemical reduction experiments by using zinc and stainless steel cathodes, respectively. Distributions of different products were observed although the reductions were not complete till the end of these experiments. It was reported that nonchlorinated organic compounds were detected when dechlorination of HCB by a liquid potassium-sodium alloy, and the dechlorination rate achieved almost 100% after a 30 min reaction [9]. However, none of nonchlorinated organic compounds was identified during our experiments. The reductive dechlorination by successive loss of chloride atoms was confirmed as the main pathway of the degradation of HCB using zinc and stainless steel cathodes. It was in agreement with the reduction studies of chlorinated compounds by the electrochemical treatment [23].

In the experiments with zinc cathode, besides HCB, five other types of chlorobenzenes were also detected, whereas 1,2,4,5-tetrachlorobenzene was the dominant byproduct. The concentrations of pentachlorobenzene, tetrachlorobenzene, and trichlorobenzene increased with time and the increase of 1,2,4-TCB was the most obvious. For the stainless steel cathode, the concentration of pentachlorobenzene, 1,2,4,5-tetrachlorobenzene, 1,2,3,4-tetrachlorobenzene, and 1,2,4-trichlorobenzene increased with time and the increase of 1,2,3-TCB was the most obvious. These experiments showed the same trend with the byproducts that was observed when Yang-hsin Shih utilized Pd/Fe bimetallic nanoparticles to reduce HCB [8].

#### 3.3.2. Graphite and RuO_2_/Ti Cathodes

**Figure 4:**
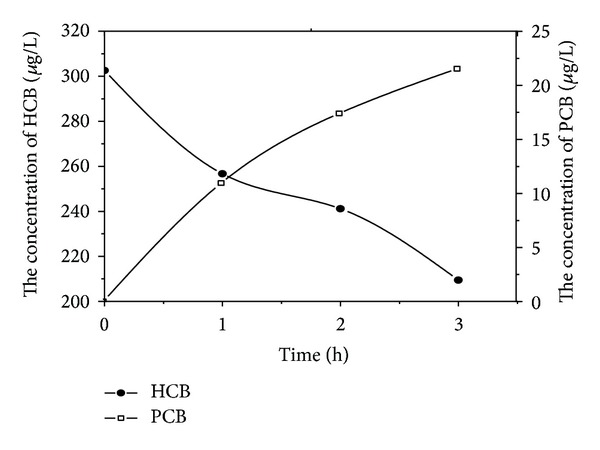
Variation of HCB and its dechlorination intermediates concentration using graphite cathode.

Figure 4 showed that the removal efficiency of HCB was 30.8% after 3 h when the graphite was utilized as the cathode, and pentachlorobenzene was the only detected degradation product. Moreover, the removal efficiency of HCB was only 10.4% after 3 h when RuO_2_/Ti electrodes were both used as the cathode and anode. In addition to pentachlorobenzene, 1,2,3,5-tetrachloro benzene was also observed as degradation product.

**Figure 5:**
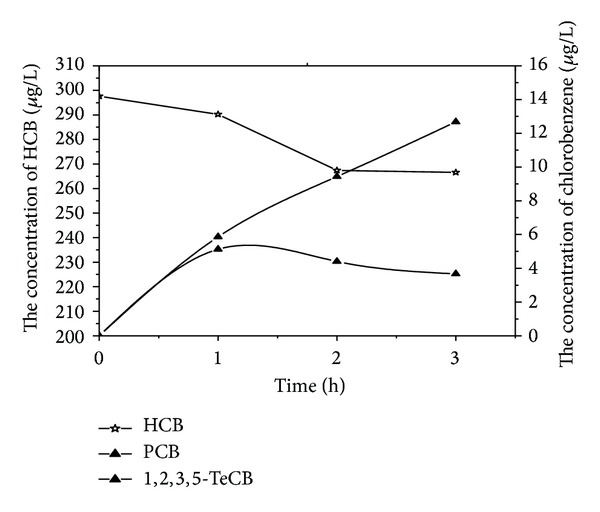
Variation of HCB and its dechlorination intermediates concentration using RuO_2_/Ti cathode.

It could be seen from Figure 5, that the electrochemical reduction potential of zinc and stainless steel cathodes was higher than that of graphite and RuO_2_/Ti cathodes.

### 3.4. Analysis of HCB Electrochemical Degradation Mechanism

The oxidation process was reported in the treatment of chlorinated organic contaminants with zero-valent iron in oxygen-rich conditions and photocatalytic oxidation [24, 25]. In our experiment, however, none of the oxidation productions was tested during the electrochemical degradation of HCB. Thus the degradation process of HCB was mainly reductive dechlorination and occurred on the cathode.

The analysis on the intermediates of HCB dechlorination with different cathode materials indicated that, for the electrochemical degradation of HCB, different cathode materials may affect not only the removal efficiency of HCB, but also the degree of reductive dechlorination. It should be underlined that the mechanism of the reduction reaction could be different for the different electrodes, depending on the possible different reaction intermediates.

The reason for electrochemical reductive dechlorination of chlorinated organic pollutants was that hydrogen atoms with chemical adsorption were generated on the electrode surface by water electrolysis and then the hydrodechlorination reaction followed. The basic steps were shown as follows:

1. 2H_2_O + 2e^−^ + M → 2(H)_ads_M − 2OH^−^
2. R − Cl + M → (R − Cl)_ads_M
3. (R − Cl)_ads_M + 2(H)_ads_M → (R − H)_ads_M + HCl
4. (R − H)_ads_M → R − H + M

Hydrogenolysis depends on the first step, that is, whether the electrode can effectively adsorb the hydrogen atoms generated by electrolysis, and the third step was the key step of the dechlorination reaction. In the experimental process, pH value of the cathode chamber was observed to have a rapid increase to be alkaline, which was consistent with the above mechanism.

Regarding 1,2,3-TCB as a typical representative of persistent organic pollutants, Kumiko Miyoshi et al. [10] selected different cathode materials including RuO_2_/Pt/PdO, Pt/IrO_2_/RuO_2_, RuO_2_, PdO, Pt, PdO/Pt, Pd/Pt, Pt, and Pd to study the dechlorination process. By testing the intermediate dichlorobenzene of the step-by-step dechlorination, it was found that different cathode materials have different dechlorination paths; it was mainly metadechlorination by Ru, Pd cathode, while it was mainly orthodechlorination by Pt cathode.

**Figure 6:**
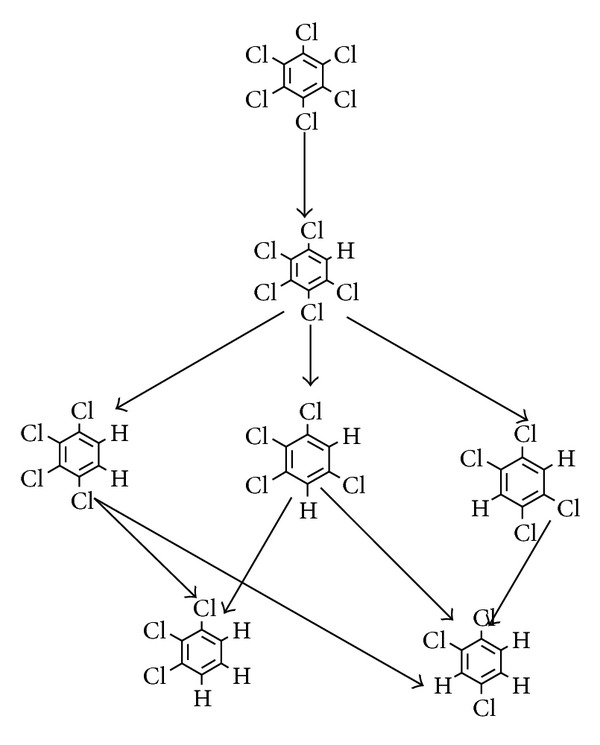
The proposed degradation pathway of HCB on stainless steel cathode.

In terms of the chemical activities of the electrode materials, it was found that Zn was the most active and then followed by stainless steel, graphite, and RuO_2_/Ti. The electrons that reach the Zn cathode in the electrolytic process can be release more quickly which are conducive for cathode materials to be more active. Atomic hydrogen has strong reducibility, and it can replace the chlorine atoms on HCB, pentachlorobenzene, and tetrachlorobenzene. The possible reaction path when graphite was used as cathode is given in Figure 6.

Analysis on the gas chromatography of HCB electrochemical products utilizing different cathode materials showed that cathode material can significantly affect the dechlorination extent of HCB. In this process, chlorine atoms were replaced one by one various intermediates such as pentachlorobenzene, tetrachlorobenzene, and trichlorobenzene occurred. The tetrachlorobenzene could be obtained when zinc and stainless steel are utilized as the cathode.

## 4. Conclusions

Electrochemical reduction of HCB using RuO_2_/Ti anode and Zn, stainless steel, graphite, and RuO_2_/Ti cathodes in a self-made cell was studied. It was found that the degradation process of HCB was reductive dechlorination occurring only on the cathode. The removal efficiency of HCB was highly sensitive to the cathode materials and the descending order of removal efficiency was shown as follows: zinc > stainless steel > Graphite > RuO_2_/Ti. Meanwhile, gas chromatography analysis for HCB electrochemical products showed that the type of cathode materials significantly affects the dechlorination extent of HCB. In the process, chlorine atoms were replaced one by one and various intermediates such as pentachlorobenzene, trichlorobenzene, tetrachlorobenzene were detected, and the tetrachlorobenzene was obtained when zinc and stainless steel were utilized as the cathode. Rapid dechlorination of HCB suggests that the electrochemical method using zinc or stainless steel as cathode could be used for remediation of polychlorinated aromatic compounds in the environment.
